# Effects of taichi on physical and psychological health of college students: A systematic review

**DOI:** 10.3389/fphys.2022.1008604

**Published:** 2022-09-29

**Authors:** Fengmeng Qi, Kim Geok Soh, Nasnoor Juzaily Mohd Nasirudddin, Yiqiang Mai

**Affiliations:** ^1^ Department of Sports Studies, Faculty of Educational Studies, Universiti Putra Malaysia, Seri Kembangan, Malaysia; ^2^ Henan Polytechnic University, Jiaozuo, China

**Keywords:** taichi, flexibility, cardiopulmonary functions, mood, coordination theory. relaxation theory

## Abstract

**Background:** Increasing studies have documented taichi’s usefulness in physical and psychological health in various participants, especially patients or the elderly. However, there is a need for a systematic review to evaluate its effects and health benefits among college students.

**Objective:**The present study aims to evaluate the current literature surrounding the effects of taichi on physical and psychological health among college students and identify the experimental areas for future research to establish guidelines for learning and teaching taichi in university.

**Methods:** The literature search involved several databases (PubMed, EBSCOhost, Web of Science, Scopus, and China National Knowledge Infrastructure). Subsequent research utilised the Preferred Reporting Items for Systematic Reviews and PRISMA checklist. In addition, the “QualSyst” tool assessed the quality of full-text articles.

**Results:** A total of 22 articles were analysed, out of which eight were strong, and 14 were of moderate quality. It is challenging to conduct a meta-analysis since the research contents were distributed differently. The general finding demonstrated that Taichi interventions have beneficial effects on college students. For example, the physical and psychological health benefits compared to other control groups include balance, leg strength, flexibility, cardiopulmonary functions, reducing stress, anxiety, and depression, and improving attention. However, there are some uncertain parameters in a state of poor or no evidence, such as upper strength, mood, and psychoticism.

**Conclusion:** Overall, this study shows that Taichi exercise is beneficial for college students compared to the control group. Evidence of health benefits for females is more than males. In addition, the current evidence showed that the effectiveness of taichi does not match some other sports such as Pilates, Yoga, Mindfulness courses, and even bodybuilding exercise. This research analyzed the mind-body mechanism of included studies. It revealed that it is difficult for college students, as a beginner of Taichi, to regulate an even breathing and quiet mind while maintaining low posture movements. Therefore, this study assumes that relaxation theories and approaches of Taichi that are easier to understand and closer to Chinese traditional Taichi theory are more appropriate in the research trials compared with coordination theory.

**Systematic review registration:** https://www.crd.york.ac.uk/PROSPERO/, identifier CRD42021278032.

## Introduction

Taichi, also translated as *taiji*, *taijiquan*, or *Tai chi chuan*, is a mind-body exercise originating from China (Ma et al., 2012; Wang et al., 2014). Over time, this form of exercise has evolved into more than five different styles: *Chen*, *Yang*, *Sun*, *Wu* (吴), and *Wu* (武) (Physical Education Press, 1995; Yang, 2003; Ma et al., 2012). Taichi was applied in most studies as a low-moderate intensity exercise since it comprised of a series of postures linked by gentle and graceful movements (Zhou et al., 2000, 2004; Esch et al., 2007; Lee et al., 2009; Caldwell et al., 2011). Previous research showed that Taichi has significant benefits to the promotion of health, and regularly practising Taichi improves aerobic capacity, muscular strength, balance, health-related quality of life, and psychological well-being (Yeh et al., 2008; Kwok et al., 2010; Ho et al., 2014; Ghandali et al., 2017; Winser et al., 2018; Yang et al., 2018, 2021; Zhong et al., 2020). Further, past reviews have provided evidence of physical and psychological variables, such as balance, hypertension, self-efficacy, and other well-being variables (Lee et al., 2009; Wang et al., 2010, 2014; Ren et al., 2017; Lyu et al., 2018; Zhong et al., 2020; Yang et al., 2021). Taichi is now often used as a form of complementary therapy since its series of postures linked by gentle and graceful movements combined with deep breaths create no limitation to exercisers (Lee et al., 2009). Therefore, many studies on Taichi intervention and health issues by examining the effects on physical and psychological variables among various populations, in particular, special groups such as patients, the elderly, and those with obesity issues (Mihay et al., 2003; Xu et al., 2005; Wu, 2008; Kwok et al., 2010; Chang et al., 2013; Manor et al., 2013; Hawkes et al., 2014; Azimzadeh et al., 2015; Zhu et al., 2016; Cha et al., 2020; Salehian et al., 2021).

The physical and psychological health of college students is a hot concern issue as college life represents a critical transitional period in the life of young adults. The other reason so many scholars engaged in this field is that college students’ physical and psychological problems face serious situations and could influence their lives and learning. For instance, a study found that college students have a high prevalence of sedentary behavior in Pakistan, which affects their health (Salehian et al., 2021). A survey conducted in China showed that more than 10% of students who have suicidal ideation might be associated with sleep disturbance (Ge et al., 2019). This issue is not a particular case since a team also found the same conditions among United Kingdom college students (Akram et al., 2020). It was proved that Taichi exercise could improve mindfulness which is positively related to sleep quality, and eliminate the passive mood of college students (Caldwell et al., 2010; 2011). Besides that, the Taichi exercise has no limitations of area and instruments, which is too convenient for college students to change their unhealthy lifestyle.

Unhealthy lifestyle was more often considered an essential factor, the lack of physical activities is the primary cause leading to poor health (Moratalla-Cecilia et al., 2016; Velten et al., 2018; Ge et al., 2019; Jakicic et al., 2019; Ren et al., 2021). Therefore, Taichi was often used to improve physical and psychological health among various populations since its mind-body movement characteristics incorporate physical exercise and pay attention to psychological cultivation. Although previous studies have confirmed its effectiveness, the main focus was on patients, the elderly, and other special groups (Mihay L et al., 2003; Xu et al., 2005; Wu, 2008; Azimzadeh et al., 2015; Wang S.-J. et al., 2020). Previous reviews have demonstrated its effectiveness mainly on psychological issues or the overall well-being of various populations (Lee et al., 2009; Wang et al., 2010, Wang et al., 2020 D.; Schleicher et al., 2012; Lyu et al., 2018; Tong et al., 2018; Winser et al., 2018; Cheng et al., 2019; Leung et al., 2019; Zhong et al., 2020; Huang et al., 2021; Yang et al., 2021). There is no doubt that there is a lack of convincing evidence to estimate if Taichi is helpful for the physical and psychological health of college students and for students and physical education teachers to be well-informed of such benefits. The present study systematically reviewed the relationship between Taichi and physical and mental health outcomes on college students by critically assessing and summarising the details from existing studies in English and the Chinese language.

## Methods

### Search strategy

A comprehensive computerised search was conducted on the following databases: PubMed, Web of Science, Ebscohost, Scopus, and China National Knowledge Infrastructure (CNKI), from inception to 25 July 2021. In addition, Baidu Scholar and Google Scholar were also used to find articles related to this study. The search was conducted by title/abstract with a pre-defined combination of keywords, for example of PubMed (TITLE-ABS-KEY (“Taichi " OR “Tai ji quan” OR “Taiji” OR “Tai chi chuan” OR “shadowboxing”) AND TITLE-ABS-KEY (“physical health” OR “health” OR “well-being” OR “physical well-being” OR “psychological health” OR “psychological stress” OR “mental health” OR “quality of life” OR “happiness” OR “emotional health” OR “mental hygiene”) AND TITLE-ABS-KEY (“college students” OR “undergraduates” OR “university students")). Other search terms used were (“Tai-ji” or “Taichi " or “Chi, Tai” or “Tai Ji Quan” or “Ji Quan, Tai” or “Quan, Tai Ji” or “Taiji” or “Taijiquan” or “T’ai Chi” or “Taichi Chuan” or shadowboxing) AND TX (physical health or physical wellbeing or physical illness or physical health problems) OR TX (“body mass index” or “BMI” or “physical fitness”) AND (college students or university students or undergraduates or young adults).

### Eligibility criteria

The trials were conducted based on the following inclusion criteria: 1) RCTs (randomized control trials) or NRCTs (No randomized control trials), comparing Taichi (no limit on the duration, frequency, or style) with other exercises, no treatment (NT) control group (CG). 2) One group of pre-post experimental studies applied Taichi as an intervention (NRNCT: no randomized no controlled trials). 3) Previous trials with participants average older than 18 years, and average not more than 40, without any unique diseases, mental issues or disabilities, or sub-health problems (no restriction on gender, nationality, or ethnicity). 4) Trials published in Chinese and English. The eligibility criteria is shown in Table 1.

**TABLE 1:** Inclusion criteria according to the PICOS conditions.

Items	Detailed inclusion criteria
**Population**	Healthy college students
**Intervention**	Taichi
**Comparisons**	Active or non-active comparisons and single-group trials
**Outcomes**	Physical health (parameter of cardiorespiratory fitness, body composition, flexibility, muscular strength, and muscular endurance)
	Psychological health (stress, depression, anxiety, mood, attention, parameters of SCL-90, and SF36V2)
**Study designs**	RCT or Non-RCT

The exclusion criteria were: 1) The study design of articles was a cross-section, survey, investigation, protocol, feasibility report without sufficient data were all excluded. 2) Participants were not healthy college students such as patients, the elderly, those with drug addiction issues, or special students who were seriously depressed, or had sub-health issues. 3) Meeting abstracts, book sections, short communications in languages other than English and Chinese. 4) Some articles without English abstracts, deficient data, and low-quality data were also excluded.

### Study selection

The Zotero reference management software eliminated duplicates from the retrieved studies. Next, the search strategies were examined with the help of an experienced librarian. Subsequently, two independent reviewers (Qi, Mai) checked and selected all articles by titles and abstracts to identify the relevant studies. The selection and assessment of full text utilised the predetermined inclusion and exclusion criteria before the final reports entered a qualitative synthesis. Unavailable articles were excluded after the full-text reading. Finally, the criteria were checked and agreed upon for each article by the third reviewer team (Soh, Nasnoor).

### Protocol and registration

The research protocol was determined and checked for conflicting results with previous literature. The existing Taichi reviews lack the focus on college students’ physical and mental health aspects and thus, justified the innovation of the proposed protocol. The protocol of search strategy, data collection, and planned analysis of this systematic review was registered in PROSPERO (CRD42021278032): https://www.crd.york.ac.uk/PROSPERO/.

### Data extraction and quality assessment

Two reviewers extracted the data independently according to the predetermined PICO standard. The information carefully extracted for all eligible publications included first author, year of publication, research design, sample size, control group, participant characteristics (example, age, and gender), intervention features (type, length, and frequency), and research outcomes. The third team of reviewers checked the information through the standard form. Methodological quality assessments were also conducted independently by two reviewers who applied the quantitative assessment tool ‘QualSyst’ by (Kmet and Lee, 2004).

## Results

### Search results and study characteristics

**FIGURE 1:**
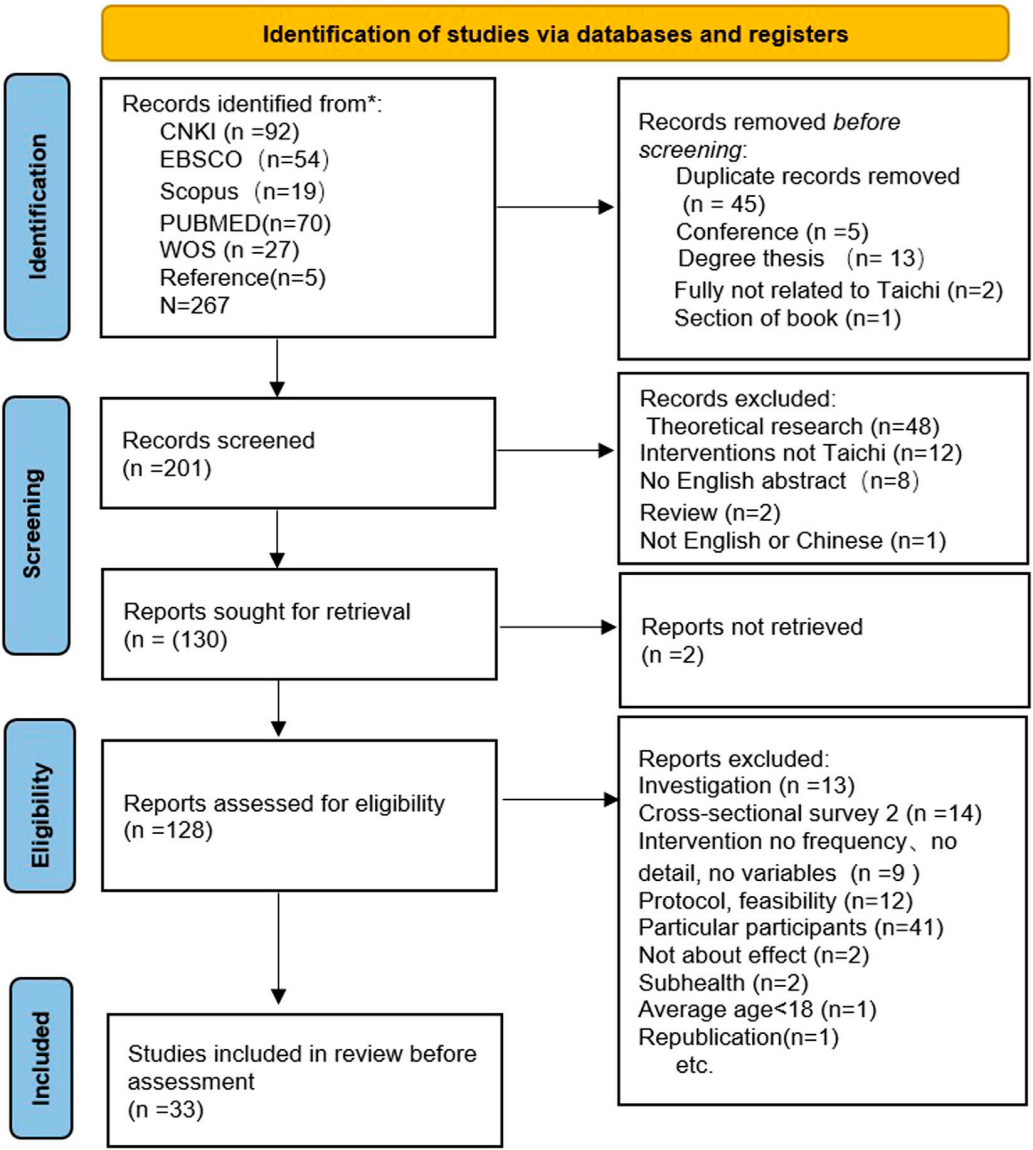
Article selection process.

A total of 262 articles were extracted and searched from databases and five studies from references. Out of the 201 articles screened, only 128 full-text articles were read after the irrelevant abstract and titles were excluded. After removing the duplicate, conference, degree thesis, studies of investigation, cross-sectional survey, protocol, feasibility, studies of data defect, and participants in non-conformity. Finally, 34 full-texts were assessed after the process of selection shown in Figure 1. Two single group studies were found with similar data in the 34 articles, and only one was retained (Wang et al., 2004; Wang, 2008). All the previous studies included in the existing research were experimental series, including 4 RCT, 18 NRCT, and 11 One group pre/post-test studies. (Figure 1).

### Study quality assessment

Quality assessment of these 33 selected articles showed that the 4 RCT and 4 NRCT articles were of solid quality, 14 reports were of moderate quality, and the balance 11 articles were of low quality. Only two studies blinded the researcher, and the allocation concealment was inadequate in all the other articles, while one study blinded their participants. Table 2 shows the total assessments of these articles. Conclusively, 22 articles were analysed in the systematic review after 11 were dropped since their quality scores did not fit the requirement (Table 3).

**TABLE 2:** Overview of included studies.

Publication	Participants	Intervention	Comparison	Measurement	Design	Outcomes
Zheng, (2017)	*n* = 200	24 style Taichi + basic skill + theory; f = 1 classes/week*5 months	Taichi only	MH: SCL-90	One group pre-post: NRNCT	Body Shape (WC, HC, Weight)↑; Physical function (RHR, LC)↑; Pyscial fitness (Stand on one foot with eyes closed↑, sit- reach↑, 800 m↑, jump↑, front kick↑); SCL-90↑
	sex = female			PH: body-composition		
	age = 18–21			Cardiopulmonary function; Balance; muscular strength		
Sui et al. (2018)	*n* = 90	style = no mention	L- physical score vs. M score, H score group	MH: Subjective Exercise Experience Scale (SEES)	Three groups pre-post: NRCT	LG and MG-PH (sit-ups↑, jump↑, RB↑,sit-reach↑, weight↔, BMI↔), HG↔ (decrease trend)
	sex = female	f = 2 s/week*4 weeks classes		PH: Japanese national physical fitness test method		MH (happiness↑; fatigue↑; troubled feeling↑)
	age = no mention (grade3-4)					
Shi et al. (2014)	*n* = 72	style = no mention	two teaching methods of Taichi; (Decomposition and Connection-Group Cooperation-Gradual Examination”) vs (standard classes)	MH: SCL-90	Two groups pre-post: NRCT	Height↔, weight↔, sit-ups↔. VC↑, VCBMI↑; Jump↑ and 800 m↑
	sex = female	1 s/week*one semester		PH: National physical and health standards for students in China		SCL-90 (F2-F5↑, F8↑)
	age = no mention (grade 1)					
Wang, (2021)	*n* = 60	style = no mention	Taichi vs. Aerobics	MH:SCL-90	Two-groups pre-post: CCT	body-building exercise VS. TC (Height↔; Weight ↑,•; BMI ↑•; WHR↔; Body fat↑; LWB↔,•; Rate of body fat•; Skinfold,↑•) (RHR↑, SBP, DBP, Cardiac function index↑, Step index↑•, LC↑•, VCBMI↑•, Maximum oxygen uptake↑, Simple reaction time↑, Stand on one foot with eyes closed↑); SCL-90(F1-F8↑, F9↔, F1, F3, F4, F5•)
	f = 60	frequency = no mention		PH: Body-composition; Cardiopulmonary function; balance; reaction time		
	age = 20–24	long = 4 months				
Robert-Mccomb et al. (2015)	*n* = 17	style = no mention. F = 60 m*2S/week*8 weeks	Taichi vs. CG	MH: stess; anxiety	Two-groups pre-post: RCT	STAI scores↑; SDNN; Coping styles (PFSOC-RF↑、PFSOC-RC↑、PFSOC-SP↑); Heart rate↑
	sex = male			PH: heart rate-stressor		
	age = 18–45					
Converse et al. (2014)	*n* = 28/44	24 style, f = 50 m*2 s/week*15 weeks	Taichi vs. CG	MH: attention; cognitive measures; ADHD; Affective processing	Two groups pre-mid/post-test: NRCT	CM↔, (WM↔, PB↔, RI↔, AP↔, ADHD SS,↔); ADHD (Inattention↑, Hyperactivity–impulsivity↑); AP (bias↑, RT variability↑)
	sex = f/t>57%			**PH: Balance. Lesuire -exercise time**		
	age = 18–34					
Zhu et al. (2019)	*n* = 125	24 style; f = 60 m*5 s/week*4 weeks	Taichi only	MH: Adolescent Self-Rating Life Events Checklist; Patient Health Questionnaire-9	One-group pre-post:NRNCT	ASLEC↑; PHQ-9 ↑
	sex = f/m (94/31)					
	age = 19.15 ± 0.87					
Hua and Sun, (2021)	n = 210	24 style; f = 80 m*1 s/week*12w	Taichi vs yoga, body-shape exercise, CG	MH: mindfulness; depression, anxiety, stress	Four-groups pre-post: CCT	MAAS(Taichi, yoga)↑; MAAS(BSE,CG)↔; Depression (Taichi, yoga)↑; Dpression (BSE,CG)↔
	f/m = 177/33					
	age = 20–22					
Zheng et al. (2015)	*n* = 206	24 style, f = 1 h*5 s/week*12w	Taichi vs CG	MH: self-esteem, stress, attention	Two groups pre-post: RCT	Taichi vs CG (self-efficacy, self-reported psychological symptoms, stress, and attention) ↔; flexibility (Sit-reach)↑; balance, (open eyes perimeter↑, closed eyes perimeter↑); SO (cardio-pulmonary function, blood pressure, HR, mood and mindfulness, self-esteem, quality of life, and quality of sleep)↔
	sex = f/m	12 weeks Follow-Up Period		PH: Balance, lower limb proprioception function; flexibility		
	age = 20.6 ± 1					
Wang, (2008)	*n* = 11/19	24 style; f = 1 h*2 s/week*3 months	Taichi only	MH: MHD of SF-36V2	One-group pre-post: NRNCT	1. PHD↔(BP↑, GHP↑)
	sex = m/f			PH: PHD of SF-36V2		2. MHD↑(RE↑, MH↑, VT↑)
	age = 24.23					
Wong et al. (2020)	*n* = 240	24 style; f = 1 h*2 s/week*18 weeks	Taichi only	MH: SCL-90	One-group pre-post:NRNCT	Well-being (PTS,PHS,BV,PA,PS)↔; Scl-90 (F1↔,F5-F7↔,F2-F4↑,F8-F9↑)
	sex = m/f (192/48)			PH: Well-being		
	age = 19–21					
Yuan et al. (2017)	*n* = 28	24 style; f = 1 h*4 s/week*20 weeks	Taichi vs CG	PH: body composition; Power cycling test; blood collection	Two-groups pre-post: CCT	(Height; Weight↑; Body fat↑; BMI↑; HR↔; SBP↑; DBP↑; LC↑; Leg strength↑; Basal metabolic rate↔); (Quiet state: E; NE; CST; NE/CST.TCVS CG,↔) (Immediately after acute motor stress: E; NE; CST; NE/CST.T VS. C,↑)
	sex = f/m (14/14)					
	age = no mention					
Nedeljkovic et al. (2012)	*n* = 70	37 *Yang* style (selected 18 movements); f = 60 min*2 s/week*12 weeks	Taichi vs CG	MH: stress; repeated salivary cortisol levels; repeated a-amylase, heart rate; depression	Two groups pre-post: RCT	Taichi vs CG: stressfulness↑, Calmness↑, Mood↔; a-Amylase↑, HR↑
	sex = f/m=(32/17)					
	age = 35.74/35.47					
Chung et al. (2013)	*n* = 48	style = *Yang* (self-made)	TCV (vibration) vs TCC, CON	PH: Balance	Two groups pre-post: RCT	Muscle activation Balance (MA↑), Balance control (KE↑,KF↔); First jump (KE↔,KF↑); Second j (KE↔,KF↔)
	sex = f/m (25/23)	30 m*3 s/week*8 weeks				
	age = 18–22					
Caldwell et al. (2009)	*n* = 98	*Chen* style Taichi; f = 50 m/3 s/week or 75 m/2 s/week*15 weeks	Taichi vs Pilates, recreational (classes)	MH: efficacy, sleep quality, mood	Three groups pre-mid-post; NRCT	Pilates (SE↑,SRE↑,insomniacs↑); Taichi (SE↑), sleep quality↔; mood↔; taichi; and Pilates (positive↑,relaxation↑, negative mood -mid↑); strength and balance,↔.
	sex = f/m50/48			PH: strength, Balance		
	Age = 18–32					
Gallego et al. (2016)	*n* = 282	Style = no mention; f = 30 m*2 s/week*12weeks	Taichi vs Mindfulness, Yoga, and CG	MH: anxiety, stress, depression	Four groups pre-post: CCT	Mindfulness > Yoga > Taichi > Control; Mindfuness (stress↑); Yoga (anxiety↑); Taichi (anxiety↑),CG↔, DASS T↔
	f/m = 154/128					
	age = 18–49					
Caldwell et al. (2010)	*n* = 166	style = *Chen*	Taichi vs Pilates, GYROKINESIS^®^ courses	MH: mindfulness; sleep, stress, quality, self-efficacy, mood	3 groups pre-mid-post: NRCT; (HLMs)	mindfulness↑; pilates > taichi > GYROKINESIS^®^; mindfulness-m-(Tired Mood, Negative Arousal, Relaxed Mood, and Perceived Stress) - sleep quality
	m/f = 25/141	f = 50 m/3 s/week*15 weeks				
	age = 18–41	or 75 m/2 s/week*15				
Gao et al. (2012)	*n* = 90	style = no mention	Taichi vs. Taichi Tuishou	MH: SCL-90	Two groups pre-post:CCT	SCL-90: Taichi (F1-F3↑); Tui shou (F1↑,F4-F6↑)
	sex = f/m (44/46)	f = 50 m*2 s/week*16 weeks				
	age = 18–22					
Cao et al. (2014)	*n* = 266	taichi classes = no style	basketball, badminton, swimming vs. Taichi	MH: SCL-90	Four-group pre-post: NRCT	All sports-MPH(2400 m↔, Jump↔, sit-reach↔, push-up↔), FPH (2000m↔, Jump↔,sit-reach↔, ↔sit-ups); SCL-90: Basketball, Badminton and swim-(F2↑, F8↑), Taichi (F1↑, F4↑, F9↑)
	sex = f/m	f=(90min + 60*2)/week*24 weeks		PH: National physical and health standards for students in China		
	age = no mention					
	
Nie et al. (2020)	*n* = 20	24 style	Taichi vs. Wuqinxi vs. CG	PH: ankle muscle strength	Three-group pre-post: NRCT	FAE-L,FAE-R, WQX vs Taichi,↑; RMAE, Taichi vs WQX,↑
	f/m = no mention	12 weeks; no frequency				
	age = no mention					
Caldwell et al. (2011)	tj = 76;;cg = 132	Chen-style taichi; f = 50 min/2 s/week*15weeks	Taichi vs special recreation	MH: mindfulness, sleep quality, self-efficacy, wellbeing, mood	A Cohort Control Study; pre-mid-post test: NRCT	Taichi vs SR (mindfulness↑•, wellbeing↑•, sleep quality↑•, self-efficacy↔)
	m/f = no mention					
	age = 18–48					
Esch et al. (2007)	*n* = 21	Traditional *Yang* style Taichi; f = 90min*1 s/week*12weeks + 12s/day (record); follow-up	Taichi only	MH: SF36	(T0-T3)4s test; longitudinal cohort study: NRNCT	PH(SBP↔,DBP↔; HR↔); Stess (PSP↔; FSC↑,PMS↑); SF36: PHD (GHP↑,BP↔,PF↔,RP↔); MHD (SF↑,VT↑,MH↑,RE↔)
	m/f = no mention			PH: Arterial blood pressure, heart rate, and free saliva cortisol		
	age>18					

Note: sig between group•; sig↑; not sig↔; SCL90 (F1 = somatisation; F2 = obsessive-compulsive; F3 = interpersonal sensitivity; F4 = depression; F5 = anxiety; F6 = hostility; F7 = phobic anxiety; F8 = paranoid ideation; F9 = psychotism); SR = special recreation; (-m-) = mediate; SE = Self efficacy; SRE = self regulatory efficacy; HR = heart rate; RHR = rest heart rate; LC = lung capacity, physical Taichi skill (PTS), physical health status (PHS), body value (BV), physical attraction (PA), physical strength (PS); RMAE = rotation muscles and evertors; FAE-R, FAE-L = flexor and extensor of right foot and left foot; ADHD = attention deficit hyperactivity disorder (Inattention, Hyperactivity–impulsivity); CM = Cognitive Measures (WM = working memory; PB = physical balance; RI = response inhibition; AP = affective processing, ADHD SS = ADHD, short screen); VC = Vital capacity; VCBMI = vital capacity body mass index; PMS-perceived mental stress; SBP-systolic blood pressure; DBP-diastolic blood pressure; SF36: PHD (body pain = BP, physical function = PF, role physical = RP; general health perception = GHP); MHD (VT = vitality; SF = social function; RE = role emotion/mental function, MH = general mental health); (PSPP) PTS-Physical taichi skills, PHS-Physical health status, BV-Body value, PA-Physical attraction, PS-Physical strength; Balance (MA = move area; KE = Knee extensor; KF = knee flexor); Lower extremity muscle power (Waist-to-Hip Ratio, WHR; lean body mass, LBM); MPH = male physical health. FPH = female physical health. LG-low, physical score group. MG-mediate score group; Mindful Attention Awareness Scale, MAASPH; Physical health (BC, body coordination; BF, Body flexibility); CF = congnitive function (AS = attention spread; PRT = passive reaction time); PSPP, Physical Self-Perception Profile; repeat bestride = RB; WC = waist circumference; HC = hip circumference; Patient Health Questionnaire-9 = PHQ-9; Adolescent Self-Rating Life Events Checklist = ASLEC., MAAS: (Score of Mindful Attention Awareness Scale); BSE(Body shape exercise group); CG (control group).

**TABLE 3:** “Qualsyst” of quality assessment.

Publication	Question described	Appropriate study design	Appropriate subject selection	Characteristics described	Random allocation	Researchers blinded	Subjects blinded	Outcome measures well	Sample size appropriate	Analytical methods	Estimates of variance	Controlled for confounding	Results reported	Conclusion supported	Ratings
								Defined and robust to bias		Well	Reported		In detail	By results?	
										Described					
(Bdel Aal Mohamed & Abdel Ghany, (2014)	2	1	1	1	N/A	0	0	0	0	0	0	N/A	1	2	L
Caldwell et al. (2009)	2	1	2	2	0	0	0	2	1	2	1	1	2	1	M
Caldwell et al. (2011)	2	2	1	2	0	0	0	2	2	2	2	1	2	2	M
Caldwell et al., 2010	2	2	1	2	0	0	0	2	1	2	1	1	2	2	M
Cao et al. (2014)	2	2	2	0	0	0	0	1	2	1	1	1	2	2	M
(Chen et al. (2006)	2	1	1	1	0	0	0	1	1	1	1	0	2	2	L
Chung et al. (2013)	2	1	2	2	2	0	0	2	2	2	2	1	2	2	H
Converse et al. (2014)	2	2	2	2	0	0	0	2	2	2	1	2	2	2	H
Esch et al. (2007)	2	1	1	1	N/A	0	0	2	1	2	1	N/A	2	1	M
Gallego et al. (2016)	2	2	2	2	1	0	0	2	2	2	1	2	2	2	H
Gao et al. (2012)	2	1	2	2	0	0	0	2	2	1	1	1	2	2	M
(Harada et al., 2018)	1	1	1	2	N/A	0	0	1	1	1	1	N/A	1	2	L
Hua & Sun, (2021)	2	1	2	2	0	0	0	2	2	2	2	1	2	2	M
(Li & Sun, 2006)	1	1	1	1	1	0	0	2	2	1	0	1	1	1	L
(Li, 2004)	1	1	1	0	N/A	0	0	0	1	1	0	N/A	0	1	L
(Li, 2007)	1	1	1	1	N/A	0	0	2	2	1	1	N/A	2	1	L
Nedeljkovic et al. (2012)	2	2	2	2	2	0	0	2	2	2	2	2	2	2	H
Nie et al. (2020)	1	1	2	0	0	0	0	1	2	2	1	1	1	2	M
Robert-Mccomb et al. (2015)	2	2	2	2	2	0	0	2	1	2	1	2	2	2	H
Shi et al. (2014)	2	1	1	1	0	0	0	2	2	1	1	1	2	2	M
Sui et al. (2018)	2	2	2	2	N/A	2	2	2	2	1	1	1	2	2	H
(Wang et al., 2007)	2	1	2	1	N/A	0	0	0	1	1	1	N/A	2	1	L
Wang, (2008)	2	1	2	2	N/A	0	0	2	2	2	1	N/A	2	2	H
Wang, (2021)	1	2	2	1	1	0	0	2	1	2	1	2	2	2	M
Wong et al. (2020)	1	1	1	2	N/A	0	0	1	2	1	1	N/A	2	2	M
Yang, (2003)	2	2	1	0	1	0	0	0	2	1	0	1	2	2	L
(Yi, (2018)	1	1	2	0	N/A	0	0	1	1	2	1	N/A	1	1	L
Yuan et al. (2017)	2	2	2	1	1	0	0	2	1	2	1	2	2	2	M
(Zhao, 2005)	1	1	1	2	1	0	0	2	2	1	1	1	1	1	L
Zheng et al. (2015)	2	2	2	2	2	2	0	2	2	2	2	2	2	2	H
Zheng, (2017)	2	1	2	1	N/A	0	0	2	2	0	1	N/A	2	1	M
(Zhou, 1998)	2	1	2	0	1	0	0	1	2	0	0	1	1	2	L
Zhu et al. (2019)	2	1	2	2	N/A	0	0	2	2	0	1	N/A	1	2	M

NA = not applicable, 2 = yes, 1 = partial, 0 = no Quality. Quality score: ≥7% high; 5% -7medium; ≥ 55% low.

### Characteristics of included studies

#### Characteristics of participants

Robert-Mccomb et al. (2015) reported stress and coping style of male college students. Sui et al. (2018), Shi et al. (2014), Wang (2021), Zheng (2017) focused on female mental and physical health (*n* = 4). Fourteen studies investigated mixed males’ and female physical and psychological parameters (Wang, 2008; Caldwell et al., 2009, 2010; Gao et al., 2012; Nedeljkovic et al., 2012; Chung et al., 2013; Cao et al., 2014; Converse et al., 2014; Zheng et al., 2015; Gallego et al., 2016; Yuan et al., 2017; Zhu et al., 2019; Wong et al., 2020; Hua and Sun, 2021). Caldwell et al., Esch et al., and Nie et al. did not deliberately distinguish between males and females in their studies (Esch et al., 2007; Caldwell et al., 2011; Nie et al., 2020). The sample size of included articles ranged from 17 to 282 participants, and only (Zheng et al., 2015) reported a sample size calculation in its protocol. There were 2,562 participants in all the 22 articles; 1447 females, 866 males, and 249 participants didnot mention their sexuality. The age of participants ranged from 18 to 49, and the most prominent mean age reported was 35.74. Some articles only registered the grade year of college students (Shi et al., 2014; Sui et al., 2018).

### Characteristics of taichi intervention

The *Yang* style Taichi was the most applied intervention since it was the earliest form of Taichi to spread across China and worldwide. Twelve articles explicitly reported the Taichi intervention of*Yang* Taichi, of which nine examined 24 styles of *Yang* Taichi (Wang, 2008; Converse et al., 2014; Zheng et al., 2015; Yuan et al., 2017; Zheng, 2017; Zhu et al., 2019; Nie et al., 2020; Wong et al., 2020; Hua and Sun, 2021). Chung et al. examined the 8-style Taichi compiled according to the *Yang* Taichi (Chung et al., 2013); Nedeljkovic et al. examined the 37 *Yang* style Taichi that taught only the first 18 movements (Nedeljkovic et al., 2012), Esch et al. explored the traditional *Yang* style Taichi, which was the second form of Taichi following *Chen* Taichi (Esch et al., 2007). Caldwell et al. examined *Chen* Taichi which was the earliest form of Taichi (Caldwell et al., 2009, 2010, 2011). However, eight studies did not mention the Taichi styles and were considered as 24 *Yang* style as explained by the search results and study characteristics above (Caldwell et al., 2010; Gao et al., 2012; Cao et al., 2014; Shi et al., 2014; Robert-Mccomb et al., 2015; Gallego et al., 2016; Sui et al., 2018; Wang, 2021). Taichi exercise cycles varied from 4 weeks to 1 year. The frequency of Taichi intervention ranged from 1 to 7 times per week, accompanied by the duration of one form of Taichi that varied from 30–90 min. The most used frequency is twice a week (*n* = 11), while 12 weeks is the most applied duration (*n* = 7), the second is 15 weeks (*n* = 5). 60 min per time is the most commonly used practising time (*n* = 8), the second is 50 min (*n* = 5), and the number of classes (*n* = 5).

### Characteristics of comparison

There were five articles designed for one group pre/post-test Taichi intervention. In addition, there were six studies designed per control group, and three articles compared with another sport such as Tuishou and Aerobics. The other articles designed three (*n* = 4) and four (*n* = 4) comparisons. Most multiple group designs focused on comparing the effectiveness of different sports classes such as Pilates, Yoga, Mindfulness classes, and basketball classes. Only one study divided the female students according to their physical fitness scores (Sui et al., 2018). The comparison results showed that the Taichi intervention was better than the control group. However, the effectiveness of Taichi was not as good as Pilates, Yoga, Mindfulness, and body-building exercises (Caldwell et al., 2009; 2010; 2011; Gallego et al., 2016; Hua and Sun, 2021; Wang, 2021). In addition, a study reported the declining trend of physical fitness among the high physical fitness group of females (Sui et al., 2018).

### Outcome measurements characteristics

Only two articles related to physical health (Yuan et al., 2017; Nie et al., 2020), and eight tested psychological health from different aspects (Caldwell et al., 2010, 2011; Gao et al., 2012; Nedeljkovic et al., 2012; Converse et al., 2014; Gallego et al., 2016; Zhu et al., 2019; Hua and Sun, 2021). On the other hand, twelve studies tested physical and mental health (Esch et al., 2007; Wang, 2008, 2021; Caldwell et al., 2009; Chung et al., 2013; Cao et al., 2014; Shi et al., 2014; Robert-Mccomb et al., 2015; Zheng et al., 2015; Zheng, 2017; Sui et al., 2018; Wong et al., 2020).

### Effectiveness of taichi on the physical health-related parameters

#### Effects of taichi on self-reported parameters of physical health

Self-reported scales of physical health-related parameters were less compared to psychological parameters. Three single group studies of the 22 articles included in this review presented outcomes on the effect of Taichi on physical health by physical health dimension (PHD) from SF-36v2 and the body well-being scale (Wang et al., 2004; Esch et al., 2007; Wang, 2008; Wong et al., 2020). PHD had four measures which were bodily pain (BP), physical function (PF), role physical (RP), and general health perception (GHP) (Wang et al., 2004; Esch et al., 2007; Wang, 2008). Body well-being (PSPP) included five dimensions which were physical Taichi skills (PTS), physical health status (PHS), body value (BV), physical attraction (PA), and physical strength (PS) (Wong et al., 2020). All subjects were of mixed gender. Two studies showed significant improvements in BP and GHP of PHD (Wang et al., 2004; Wang, 2008). The other study only found that GHP improved significantly (Esch et al., 2007). Accordingly, the body well-being was reported to increase without any significance (Wong et al., 2020).

### Effects of taichi on body shape

The body shape was an essential indicator for evaluating the physical health of university students to evaluate the bodyweight bias and body composition reasonably. Four studies assessed in this review designed body shape indicators of females (*n* = 4), such as weight, BMI, body fat, waist circumference (WC), WHR (Waist-to-Hip Ratio), LWM (Lean body mass), BF% (body fat ratio), and Skinfold (Shi et al., 2014; Yuan et al., 2017; Zheng, 2017; Wang, 2021). One article reported the significance of WC, HC, and weight change after one class time per week that lasted 5 months of Taichi practice (Zheng, 2017). On the contrary, another article found less significant weight change after Taichi class under a particular teaching model that lasted 4 months, in the same frequency (Shi et al., 2014). Recent research reported that Taichi significantly affected weight, BMI, BF%. It was interesting to note that body-building exercises had better outcomes than Taichi on weight, BMI, LWB, BF%, and Skinfold (46). In a trial that lasted 20 weeks there was a significant change in weight, body-fat, and MBI (Yuan et al., 2017).

### Effects of taichi on the physical function and health-related parameters

Physical function is the life activity exhibited by human tissues, organs, and systems within the whole body (Yuan and Huang, 2011). Ten studies, including four RCT, two CCT, two NRCT, and two single-group studies, evaluated the effect of Taichi on the physical function in this systematic review (Esch et al., 2007; Caldwell et al., 2009; Nedeljkovic et al., 2012; Chung et al., 2013; Shi et al., 2014; Zheng et al., 2015; Yuan et al., 2017; Zheng, 2017; Nie et al., 2020; Wang, 2021). Variables were related to cardiopulmonary, balance, and nervous system functions.

One RCT reported that balance (i.e., standing with an open or close eye) significantly improved. In contrast, cardio-pulmonary, blood pressure, and heart rates did not improve considerably after five times per week and 12 weeks of practice (Zheng et al., 2015). Two RCTs mentioned that heart rates significantly changed when measured forstress-coping or reactivity (Nedeljkovic et al., 2012; Robert-Mccomb et al., 2015). The last RCT specifically focused on balance and low extremity power and found that the move area and knee extensor of the TCV group (Taichi combined with vibration) were significantly different among groups. Further, the jump height improved in TCV and CON, but not the TCC group, which implied only low improvement for those who practised Taichi (Chung et al., 2013). One CCT found significant improvement both within-group and between the group on systolic pressure (SBP), diastolic blood pressure (DBP), and lung capacity (LC), but not heart rate and basal metabolic rate (Yuan et al., 2017). Another CCT compared body-building exercises with Taichi and found significant improvement of physical function parameters: RHR, cardiac function index, step-index, LC, VCBMI, Maximum oxygen uptake, standing on one foot with eyes closed, and simple reaction time in the two groups. The body-building exercises werebetter than Taichi significantly on step-index, lung capacity, and Vital mass index (Wang, 2021). Further, an NRCT applied a teaching model of Taichi that improved the VC and vital mass index compared to the control group (Shi et al., 2014), whereas the other NRCT reported less significant improvement in balance for physical performance within groups or time (Caldwell et al., 2009). One single group study showed blood pressure and HR that did not change dramatically after 90 min per time and one time per week that lasted 12 weeks (Esch et al., 2007), while the other observed RHR, LC, and balance (standing on one foot with eyes closed) changed significantly (Zheng, 2017).

### Effectiveness of taichi on fitness-test parameters

Eight studies examined in this review reported physical fitness-related parameters that were sit-reach, sit-ups (female), 800 m, 2400 m (male), 2000 m (female), push-up, jump, front kick, strength (low back, leg), and (RB) repeat bestride (Caldwell et al., 2009; Cao et al., 2014; Shi et al., 2014; Zheng et al., 2015; Yuan et al., 2017; Zheng, 2017; Sui et al., 2018; Nie et al., 2020). The studies’ design was one RCT, five NRCT, one CCT, and one NRNCT. The RCT showed single flexibility with sit-reach that was significantly improved (Zheng et al., 2015). Zheng conducted a one-group study and observed the improvement of sit-reach, 800m, jump, and front kick after 5 months and one time per week of class training. However, an uncertain bias was that the basic skills training may have affected the effects of Taichi since basic skills is a part of martial arts (Zheng, 2017). A significant improvement of leg strength was observed in a CCT study by the Yuan team both within-group or between groups of which the practising time of the study was 1 hour per time and four times per week that lasted 20-weeks (Yuan et al., 2017). One NRCT compared the ankle muscle strength between those who practiced Taichi and Wuqinxi. The rotation and evertors muscle strength of the ankle muscle was significantly improved by Taichi, whereas the flexor and extensor of both feet were significantly improved by Wuqinxi (Nie et al., 2020). Caldwell conducted an NRCT between Pilates and Taichi, and examined the lower back and leg strength but did not observe any significant change (Caldwell et al., 2009). One NRCT observed two-semester classes of different sports, including Taichi, that also failed to find any significant improvement in physical fitness. The time of physical classes was 90 min per time and one time per week, which lasted 12 weeks a semester (Cao et al., 2014). The other two NRCT reported improvement of different parameters. Sui tested females’ sit-ups, jump, RB, and sit-reach compared to the control group and only observed an improvement in jumps and 800 m (Shi et al., 2014).

### Effectiveness of taichi on the psychological parameters

Nineteen articles examined in this review reported psychological health-related parameters of different aspects (Esch et al., 2007; Wang, 2008; 2021; Caldwell et al., 2009; 2010, 2011; Gao et al., 2012; Nedeljkovic et al., 2012; Cao et al., 2014; Converse et al., 2014; Shi et al., 2014; Robert-Mccomb et al., 2015; Zheng et al., 2015; Gallego et al., 2016; Zheng, 2017; Sui et al., 2018; Zhu et al., 2019; Wong et al., 2020; Hua and Sun, 2021). Psychological health variables were mainly mental health tested by SCL-90 or SF36V2, depression, mood, stress, inattention, mindfulness, self-esteem, quality of life and sleep, self-efficacy, and anxiety.

### Taichi and mental health tested by SCL-90, SF36V2

SCL-90 and SF36v2 are the two most commonly used tools in mental health testing. Six studies examined in this review applied SCL-90 to test college students’ mental health. These six articles included two CCT (Gao et al., 2012; Wang, 2021), two NRCT (Cao et al., 2014; Shi et al., 2014), and two single group studies (Zheng, 2017; Wong et al., 2020). There were nine dimensions of SCL-90 that were F1-somatization, F2-obsessive, F3-interpersonal sensitivity, **F4-depression,** F5-anxiety, F6-hostility, F7-phobic anxiety, F8-paranoia ideation, and F9-psychoticism. One CCT reported that F1 to F3 dimensions was declined significantly after 90 min of Taichi class two times per week, with 60 min of extracurricular activities each time, which lasted two semesters (Gao et al., 2012). The other CCT reported a significant change in F1 to F8 dimensions after body-building exercises and Taichi intervention. There was a substantial difference between the two groups in F1, F3, **F4**, and F5 measurements that showed body-building exercise intervention was better than Taichi (Wang, 2021). One NRCT observed a significant decrease of F2, F3, **F4**, F5, F8 dimensions by group cooperation teaching method (Shi et al., 2014), whereas the other observed that F1, **F4**, and F9 sizes significantly decreased after Taichi intervention (Cao et al., 2014). Zheng reported that all dimensions significantly changed after one class time per week that lasted 5 months of Taichi intervention (Zheng, 2017). However, the other study only observed the effect on F2, F3, **F4**, F8, and F9 dimensions (Wong et al., 2020). The SF36v2 scale had eight sizes, including physical and mental health. The cognitive health-related dimensions were social function (SF), role mental/emotion function (RE), vitality (VT), and perceptions of mental health (MH). Three single group studies applied SF36V2 scales to test MHD. Two studies reported no significant change in social functions (Wang et al., 2004; Wang, 2008). The other article only reported a non-significant change in the RE (Esch et al., 2007).

### Effects of taichi on positive and negative psychological health-related parameters

The included studies in this review tested positive and negative psychological health parameters such as depression, anxiety, stress, mood, quality of life, sleep quality, self-efficacy, and attention. This study reported these parameters together since it was difficult to extract data to form different categories. Eleven articles related to these parameters were three RCT, two CCT, and six NRCT (Caldwell et al., 2009; 2010; 2011; Nedeljkovic et al., 2012; Converse et al., 2014; Robert-Mccomb et al., 2015; Zheng et al., 2015; Gallego et al., 2016; Sui et al., 2018; Zhu et al., 2019; Hua and Sun, 2021).

One RCT reported no significant effect on self-efficacy, self-reported psychological symptoms, stress, and attention after 1 h for five times per week that lasted 12 weeks for the 24 style Taichi intervention (Zheng et al., 2015). The other two RCTs focused on stress arising from different stressors such as public speaking. Nedeljkovic tested stress reactivity and observed a significant change in stressfulness and calmness, a-Amylase, but not mood (Nedeljkovic et al., 2012); and another RCT tested four times of heart rate variability (HRV) before and after two different stressors. They observed the suppressive coping style from the Taichi group (Robert-Mccomb et al., 2015). One CCT compared Taichi, Yoga, body-shape exercise, and CG, and observed that mindfulness and depression significantly changed through Yoga and Taichi intervention, while the other two groups showed no significant differences (Hua and Sun, 2021). The other CCT compared the effects of mindfulness-based cognitive therapy (MBCT), Yoga, and Taichi on stress, anxiety, and depression; and observed significant effectiveness of MBCT on stress; while Yoga and Taichi reduced anxiety. The total score of DASS (Depression Anxiety Stress Scales) indicated that MBCT had a better effect, followed by Yoga, and Taichi, respectively (Gallego et al., 2016).

One NRCT studied the inattention of healthy young adults and observed a significant difference between Taichi and CG on inattention of ADHD and affective processing bias, but not on cognitive measures (Converse et al., 2014). A team from China focused on medical students’ mental health. They applied the Adolescent Self-Rating Life Events Checklist (ASLEC) and Patient Health Questionnaire-9 (PHQ-9) to pre/post-test students’ mental health and observed a significant decrease in both total scores that showed a positive correlation between life events and mental health (Zhu et al., 2019). Caldwell compared the effects of Pilates, Taichi, and Special recreation on self-efficacy, sleep quality, and mood. They observed that Pilates and Taichi significantly improved self-efficacy, positive mood scores, and relaxation scores, but there was no substantial improvement in sleep quality. Negative mood scores decreased significantly during the semester and recovered at the end of the semester (Caldwell et al., 2009). Subsequently, they conducted a study to measure the development of mindfulness in college students through Taichi, Pilates, and GRROKINESIS courses and observed whether it affected self-efficacy, mood, stress, and sleep quality. They found the three courses significantly increased the total mindfulness scores and improved mindfulness mediated sleep quality by tired mood, negative arousal, relaxed mood, and perceived stress (Caldwell et al., 2010). The team also conducted the third study that examined mindfulness, well-being, and quality of sleep in college students with a significant increase in total mindfulness only in the Taichi group. Meanwhile, the rise in mindfulness significantly correlated with the improvement of well-being measures and sleep quality (Caldwell et al., 2011). The last NRCT measured the subjective exercise experience of females after Taichi intervention and found that it only significantly improved the happiness dimension, while the low physical fitness group experienced considerable progress in psychological well-being after participating in Taichi exercises (Sui et al., 2018).

## Discussion

The present systematic review attempts to provide current knowledge of the effects of Taichi on physical and psychological health among healthy college students. This study involved 22 trials, and 14 studies involved physical function, physical fitness, and body composition. In addition, 20 articles related to psychological health, i.e., quality of life, sleep quality, self-efficacy, stress, anxiety, depression, and mood issues. The significant data findings showed that Taichi could improve physical and psychological health among college students compared to the control group, especially psychological health and balance, flexibility. However, the current evidence showed that Taichi intervention is not as good as mindfulness courses, Yoga, Pilates, and body-building exercises among college students.

### Evaluation of participants

The evidence of the 22 trials showed that most studies focused on mixed genders (17) or females (*n* = 4), and only one article focused on male students’ stress cope reactivity. The number of males in the 22 studies was half of the number of females. Therefore, there is a lack of studies focused on males, and no studies compared the effects between females and males. In addition, one study caused the reflection of the team of this systematic review that the high fitness score group had a declining trend in physical fitness after the Taichi intervention (Sui et al., 2018). More studies are required to examine if this trend exists in male students since male students’ fitness is better than females on average.

### Evaluation of the application of taichi intervention measures and theories

There were mainly two types of Taichi applied in the selected 22 studies: *Chen* Taichi (*n* = 3) and *Yang* Taichi (*n* = 12), and seven articles did not mention any Taichi style. 24 *Yang* style, also called simplified Taichi, was mainly applied (*n* = 9) by scholars. This type of Taichi represented simplified movements and postures to learn efficiently and spread widely (64).

Taichi intervention was considered a mind-body exercise since the Taichi “Three in One” theory explained Taichi movements need to be integrated by mental concentration, balance shifting of body weight, muscle relaxation, and breath control (Caldwell et al., 2010; 2011; Zheng, 2017). Nonetheless, Wayne et al. (Wayne and Kaptchuk, 2008) deemed Taichi a complex multi-component intervention as the exercise integrated numerous physical, cognitive, and ritualistic components. Indeed, the “Three in One” theory, created from “health qigong,” described a high-level qigong state and was not for Taichi beginners.

On the other hand, many studies selected in this review mentioned that the intervention applied more than 10 years of experienced Taichi coaches to teach relaxation and breathing technology but did not describe their methodology (Esch et al., 2007; Caldwell et al., 2010; 2011; Zheng et al., 2015). One study that compared Taichi and Tuishou, an imitation of Taichi fighting, observed the greater effectiveness of Tuishou (Gao et al., 2012). Many comparisons of Taichi with other sports failed to find better validity, such as not being as good as Yoga, Pilates, body-building exercises, and mindfulness courses (Caldwell et al., 2009; 2010; Hua and Sun, 2021; Wang, 2021). Another study also found high physical score students with decreasing trend of physical health indicators (Sui et al., 2018). Why the intervention of Taichi always couldn’t reach the expected results? It was suggested that Taichi had been misunderstood and wrongly practiced (Wang, 2017). The misunderstanding and wrong practice was mainly manifested as follows: First, Taichi exercise calls for mental concentration by following the inner feeling of body movemenets rather than modulate. Because, actively regulation of mind may generate more thoughts and be lost in various fancies and conjectures. Secondly, regulating breathing could work at the beginning of Taichi exercise, but it can’t last since the body will control breathing itself when more oxygen is needed caused by low posture movements. Thirdly, low posture movements of Taichi exercise cause muscular tension, Without methods to cope this, participants will stand up to or persist in enduring muscle tension. Standing up will transform Taichi into a slow, soft, and light exercise which effect was not expected. On the contrary enduring muscle tension will break the even breathing or mind concentration which always only affects on balance by improving low limb strengthen. Therefore, the reasonable theory should be applied, especially the relaxation theory, a pivotal point in Taichi practice since it can be seen in every Taichi book, textbook, and doctrines (Physical Education Press, 1995; Chen and Silberstorff, 2012; Wayne, 2013). Chengpu Yang, the master of Yang Taichi, said, “the Taichi kungfu is relaxing, relaxing, and relaxing.” The relaxation method could eliminate the muscle tension, and the mind only needs to feel and help the relaxation of the body and mind by exhaling sometimes. In other words, all Taichi exercises only do one thing-relax. There is no misunderstanding and wrong practice. Therefore, the importance of the relaxation theory fell into neglect in most experimental studies. Accordingly, there should be more studies on the relaxation theory to explain the understanding of physiological knowledge. In addition more details should be described of Taichi intervention in future studies to be followed and repeated by readers.

### Effects of taichi on physical health

Physical health, also called health-related physical fitness, can be divided into five components: cardiorespiratory fitness, body composition, flexibility, muscular strength, and muscular endurance (Dwyer and Davis, 2007). Notably, most included studies have only applied one parameter to represent one component, such as flexibility only tested by sit-reach. Therefore, more parameters should be involved in future studies to find more evidence of Taichi on physical fitness or improve Taichi intervention.

Four studies of selected articles in this review applied the self-report scale. Three of them involved SF36v2 and observed a total improvement of all dimensions of PHD, although improvements of all the four dimensions did not appear in the same article (Wang et al., 2004; Esch et al., 2007; Wang, 2008). One study observed that all physical well-being parameters improved but were not significant: PTS, PHS, BV, PA, and PS (Wong et al., 2020). To some extent, the improvement of self-report parameters showed that Taichi exercise is beneficial. In other words, Taichi improved body perception, as described by Dr. Yong: “the theory of Taichi is not a mysterious abstract symbol, but an internal experience that can be perceived in practice” (Yi, 2018).

Eight studies reported results of cardiorespiratory fitness. Lung capacity significantly improved in four articles (Shi et al., 2014; Yuan et al., 2017; Zheng, 2017; Wang, 2021). Three studies found the cardiovascular system was improved as follows, RHR (Zheng, 2017; Wang, 2021), SBP, DBP (Yuan et al., 2017; Wang, 2021), Cardiac function index, and Step index (Wang, 2021). Two studies found that HR significantly decreased after Taichi intervention when coping with the stressor (Nedeljkovic et al., 2012; Robert-Mccomb et al., 2015). On the contrary, the RCT did not give evidence of significant improvement of cardiorespiratory (Zheng et al., 2015). Pairwise, one single-group study found no substantial change in SBP, DBP, and HR (Esch et al., 2007). Cardiorespiratory fitness is one parameter that confused researchers for various reasons. Taichi is an aerobic exercise but does not stimulate heart rate and vital capacities like other aerobic exercises like jogging or brisk walking. A study reported that the intensity of practicing Taichi was similar in different age and gender groups (Lan et al., 2012). The evidence of the effect of Taichi on cardiorespiratory was insufficient since there were no more RCTs to support such a finding.

Eight studies provided outcomes of the other four components of physical fitness: muscular endurance, muscular strength, body composition, and flexibility. The articles observed significant changes, such as balance (Caldwell et al., 2009; Chung et al., 2013; Zheng et al., 2015; Zheng, 2017; Wang, 2021), sit-reach (Zheng et al., 2015; Zheng, 2017; Sui et al., 2018), 800 m (Shi et al., 2014; Zheng, 2017), jump (Shi et al., 2014; Zheng, 2017; Sui et al., 2018), front kick (Zheng, 2017), body fat (Yuan et al., 2017; Wang, 2021), BMI (Yuan et al., 2017; Wang, 2021), leg strength (47, 53), weight (Yuan et al., 2017; Zheng, 2017; Wang, 2021), WC (Zheng, 2017), HC (Zheng, 2017), sit-ups (Sui et al., 2018), RB (Sui et al., 2018), and skinfold (Wang, 2021). The less significant improvements reported were weight (Shi et al., 2014; Sui et al., 2018), sit-ups (Shi et al., 2014), WHR (Wang, 2021), LWB (Wang, 2021), and BMI (Sui et al., 2018). One NRCT also did not find any significant improvement in physical fitness after a 12-weeks Taichi course (Cao et al., 2014).

The envidences of Taichi on physical fitness are confused as some indicators were not significant. The self-reported results were better than actual physical fitness measurements. The cardiovascular system index could not determine the benefits for college students primarily because of the results of RCT. Most NRCT and single-group studies proved that Taichi could improve body composition, balance, flexibility, speed-endurance, and explosive force, except for one NRCT. Therefore, it is necessary to consider why the differences occurred in research results. The difficulty of integrating breathing, posture, and calm minds may be the main reason (Wayne and Kaptchuk, 2008).

### Effects of taichi on psychological health

This review assessed psychological health based on 20 out of the 22 studies. However, only one RCT focused on male students’ stress and found significant changes in STAI scores. This finding suggested that the Taichi group had learned to manage their subjective reactions to a stressor (Robert-Mccomb et al., 2015).

Four studies included in this review presented inferences about the effect of Taichi on female students’ psychological health. One of these studies applied MHD of SEES. The study found that all groups (H, M, L score) recorded significant changes despite the physical score (Sui et al., 2018). The other three articles reported the SCL-90 scores as follows: 1) all dimensions and total scores significantly changed after Taichi training (Zheng, 2017); 2) only F9 did not significantly change (Wang, 2021); and 3) F1, F6, F7, and F9 did not significantly change after the one semester Taichi course (Shi et al., 2014). The results of these studies appeared to be non-unified and stable.

Fifteen studies in this review tested college students’ mental health with mixed genders. Only one of these studies focused on the inattention of healthy young adults and found the self-report score of attention improved (Converse et al., 2014). One tested medical students’ mental health by ASLEC and PHQ-9 and observed a significant change in the total score; some pressure factors (the learning pressure factor and the adaptation factor) were positively associated with the PHQ change (Zhu et al., 2019). Three of these studies reported mental health dimensions by SF36V2; the detailed results of significant changes were as follows: MHD: SF (Esch et al., 2007), RE (Wang et al., 2004; Wang, 2008), MH (Wang et al., 2004; Esch et al., 2007; Wang, 2008), and VT (Wang et al., 2004; Esch et al., 2007; Wang, 2008). Three articles applied SCL-90 to test the students’ mental health. The significant changes were as follows: F1 (Gao et al., 2012; Cao et al., 2014), F2 (Gao et al., 2012; Wong et al., 2020), F3 (Gao et al., 2012; Wong et al., 2020), F4 (Cao et al., 2014; Wong et al., 2020), F5-F8 (Wong et al., 2020), and F9 (Cao et al., 2014; Wong et al., 2020). The other studies focused on both positive and negative mood aspects. The significant changes are given as follows: mindfulness (Caldwell et al., 2010, 2011; Hua and Sun, 2021), depression (Hua and Sun, 2021), stress (Nedeljkovic et al., 2012; Hua and Sun, 2021), calmness (Nedeljkovic et al., 2012), self-efficacy (Caldwell et al., 2009), anxiety (Gallego et al., 2016; Hua and Sun, 2021), sleep quality (Caldwell et al., 2011), well-being (Caldwell et al., 2011). However, the RCT reported no significant change in mood and mindfulness, self-esteem, quality of life, and sleep quality (Zheng et al., 2015). Further, one NRCT did not observe a significant change in self-efficacy (Caldwell et al., 2011).

Although the RCT did not find a significant change in psychological parameters, a good improvement occurred after the Taichi intervention (Zheng et al., 2015). All other articles gave acceptable evidence of the effectiveness of Taichi on different aspects of psychological parameters. In particular, the negative psychological parameters were significantly ameliorated by Taichi interventions such as depression, stress, and anxiety.

### Evaluation of the mind-body mechanism

**FIGURE 2:**
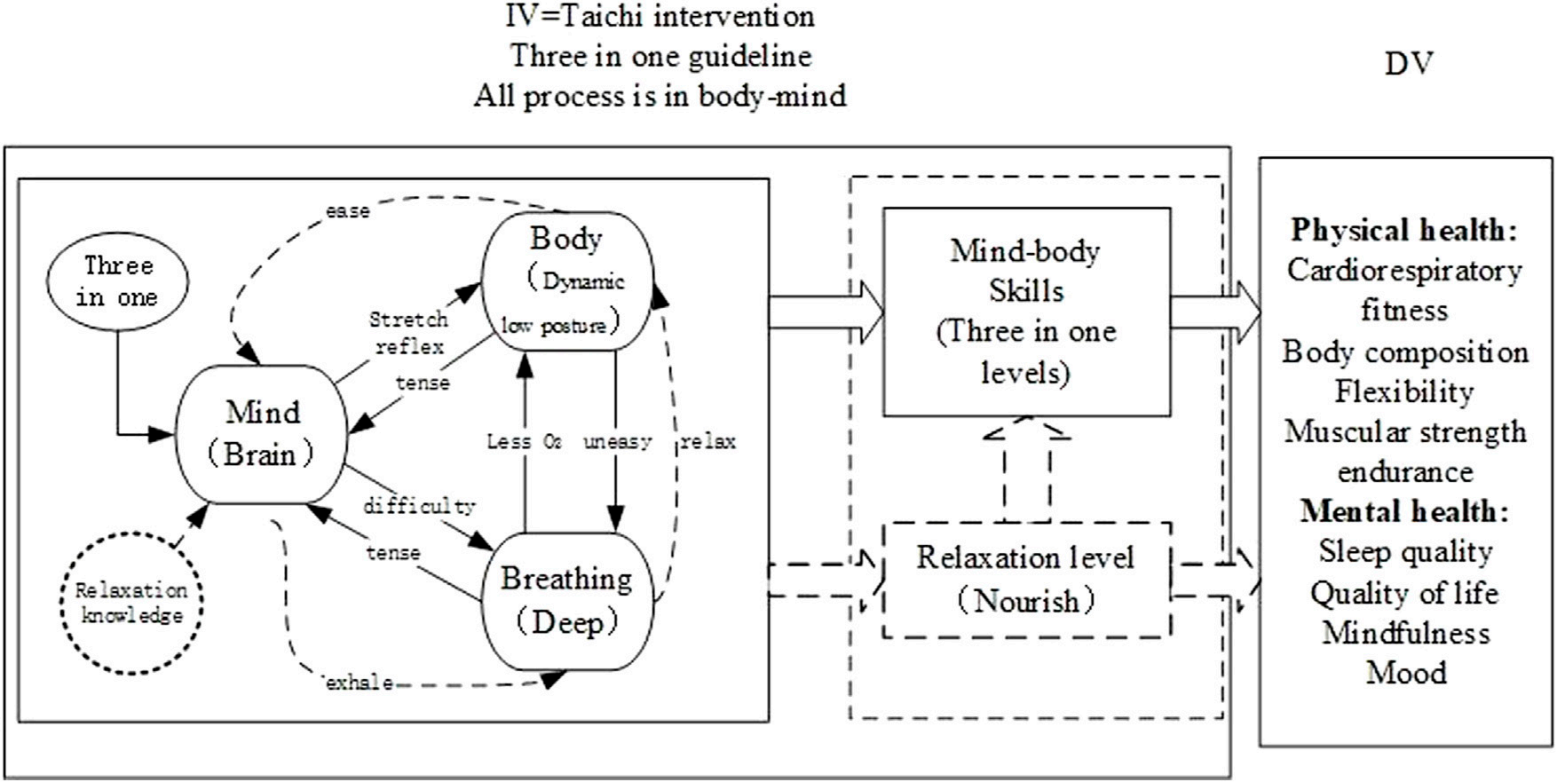
Mind-body mechanism.

The mind-body mechanism of Taichi has been explained in the “Three in One” theory, which means the practice of Taichi should integrate body movements, deep breathing, and mind concentration (Caldwell et al., 2010; 2011; Zheng, 2017). However, this theory stemmed from healthy qigong, which is not suitable for Taichi as the body movements in Taichi are not the same as health qigong (Si and Zhang, 2014). The analysis in the present study showed that the low posture movements of Taichi inevitably caused muscular tension as the leg needs to support the whole body. The mind would be nervous since the signal from the nerve conduction is muscle tension information. Therefore, the respiratory system would be forced to work for more oxygen and power. As a result, the mind, respiratory, and muscular systems cannot be integrated. As such, the knowledge and practice methods of relaxation appears crucial to the practice of Taichi. The ease of the leg can eliminate the stretch reflex and reverse the tension situation within the body (Tsatsouline, 2002). Finally, a harmonious physical and mental mechanism can be formed with the help of relaxation (Figure 2). Therefore, future studies should focus on the relaxation theory that emphasised by Taichi experts.

## Limitations

Finally, the present review provides specific evidence of acceptable quality and the beneficial effects of Taichi on physical and psychological health among college students. Nonetheless, there were several limitations due to the quality and design of Taichi articles. Firstly, there was no evidence to prove the effective frequency and lasting time since some low frequency with short time intervention reported significant changes. Pairwise, some high frequencies with a long time observed no significance. Secondly, there was a lack of high-quality, rigorous, and prospective RCTs. Thirdly, most studies focus on mixed-gender and females, and a lack of trials that focused on male college students was a weakness of the generalization of findings. Fourthly, Most studies lack the discussion of mechanism and principle. Accordingly, it was challenging to conduct any repeated research that may have caused inconsistent results in previous studies. Finally, few studies included in this review stated the sample size calculation method if such studies included an unreasonable quantity of samples that may cause bias in interpreting results (Bhalerao and Kadam, 2010).

## Conclusion

Taichi has been widely applied as a mind-body exercise for college students to promote physical and psychological health in many countries (Esch et al., 2007; Caldwell et al., 2009; Nedeljkovic et al., 2012; Bdel Aal Mohamed and Abdel Ghany, 2014; Robert-Mccomb et al., 2015; Zheng et al., 2015). However, a thesis article reported that more than 50% of students thought Taichi was only effective for the elderly (Chai, 2015). In other words, they did not feel the benefits of practicing Taichi. Therefore, this systematic review comprehensively examined the effects of Taichi on improving physical and psychological health parameters in healthy college students. In general, evidence showed that Taichi is beneficial for college students compared to the control group and the evidence of benefits in females outweighs males. The psychological benefits for college students are far more than physical fitness. The improvements of the physique index are better than the function index. In detail, the present systematic review provided strong evidence that Taichi enhanced balance, flexibility, and body composition for physical health-related parameters. Pairwise there was moderate evidence for muscular strength and self-reported parameters. There was insufficient evidence on muscular endurance and cardiorespiratory fitness. Further, there was no evidence related to the upper back and limb. In terms of psychological health, substantial evidence proved that Taichi improved mindfulness and decreased stress, anxiety, and depression. In addition, there was also moderate evidence that Taichi improved sleep quality, attention, and reduced self-reported psychological symptoms. Finally, there was poor evidence that Taichi intervened in mood and psychoticism.

Further trials on Taichi related to college students’ health should focus on male students or the differences between males and females. In addition, future studies should include high-quality, rigorous, and prospective RCTs with appropriate control groups and multidimensional dependent variables. Notably, Taichi interventions must be precisely characterised and detailed for meaningful comparisons and the convenience of repetition. In order to discover the profound potential of Taichi’s role, influential theories and guidelines should be combined in such studies. For instance, the “Three in One,” theory which aimed to integrate breath, mind, and movements, appeared insufficient and easily misunderstood among college students. However, the relaxation theory which was easy to understand, practice, and often emphasised by masters, was actually ignored since it failed to focus on exercise intensity and was difficult to explain the outcomes. Other theories about Taichi may lead to new complementary and alternative medical approaches to promote physiological and psychological effects, handle chronic medical conditions, and further illustrate the mechanisms of successful mind-body medicine.

## Data Availability

The original contributions presented in the study are included in the article/Supplementary Material, further inquiries can be directed to the corresponding author.
